# Biochemical and Histo-Anatomical Responses of *Lavandula angustifolia* Mill. to Spruce and Beech Bark Extracts Application

**DOI:** 10.3390/plants9070859

**Published:** 2020-07-07

**Authors:** Corneliu Tanase, Ruxandra Ștefănescu, Béla Darkó, Daniela Lucia Muntean, Anca Corina Fărcaş, Sonia Ancuţa Socaci

**Affiliations:** 1 Department of Pharmaceutical Botany, “George Emil Palade” University of Medicine, Pharmacy, Sciences and Technology of Târgu Mureș, 38 Gheorghe Marinescu Street, Târgu Mureș, 540139 Mureș, Romania; bela.darko@umfst.ro; 2 Department of Pharmacognosy and Phytotherapy, “George Emil Palade” University of Medicine, Pharmacy, Sciences and Technology of Târgu Mureș, 38 Gheorghe Marinescu Street, Târgu Mureș, 540139 Mureș, Romania; 3 Department of Analytical Chemistry and Drug Analysis, University of Medicine, Pharmacy, Sciences and Technology, 540139 Târgu Mureș, Romania; daniela.muntean@umfst.ro; 4 Department of Food Science, Faculty of Food Science and Technology, University of Agricultural Sciences and Veterinary Medicine, 400372 Cluj-Napoca, Romania; anca.farcas@usamvcluj.ro (A.C.F.); sonia.socaci@usamvcluj.ro (S.A.S.)

**Keywords:** bark extracts, beech, bioactive compounds, lavender, spruce, volatile compounds

## Abstract

This paper aims to assess the biological responses of *Lavandula angustifolia* Mill. to beech and spruce bark crude extract application. Thus, the biological activity of bark extracts was assessed by determining the germination capacity, biomass production, histo-anatomical aspects and photo-assimilatory pigment accumulation, and by quantitative and qualitative volatile compounds analysis. The application of spruce bark extract (500 mg dry bark/100 mL solvent) resulted in a better development of the leaf tissue and an increase in foliar biomass. We observed the stimulating effect of photo-assimilating pigments accumulation, for all experimental variants, compared to the control. Also, the amount of volatile oil was significantly higher in the treated plants with spruce bark extract (500 mg dry bark/100 mL solvent). Some volatile compounds (cyclen, borneol, cryptone, santalen, and caryophyllene oxide β—farnesene) were identified only in the experimental variants. Also, in the experimental variants, an increase in the quantity of limonene, linalyl acetate and lavandulol was observed. These preliminary results showed that the beech and spruce bark extracts can have biological activities and influence the production of volatile oil in *Lavandula angustifolia*, causing significant changes in the phytochemical profile of the essential oil. Thus, forest waste bark extracts could be recommended as natural bioregulators in lavender crops.

## 1. Introduction

Lavender is a globally well-known aromatic and medicinal herb from the Lamiaceae family. The essential oil obtained through distillation is the main product used from this plant. Its unspecific condition for cultivation has increased the agricultural production of lavender, and it is considered to be a sustainable crop. Although many lavender species exist, only three are considered to be important sources of lavender oil: the genuine lavender—*Lavandula angustifolia* Mill. (sin. *Lavandula officinalis* Chaix), spike lavender—*Lavandula latifolia* Mill. and lavandin—*Lavandula hybrida* Revr.

Many utilizations of lavender essential oil are based on empirical data, but in recent years, the essential oil has gained considerable attention due to its therapeutic effects demonstrated by in vitro and in vivo studies [1,2]. Recent research has shown that lavender essential oil has beneficial effects on anxiety, depression and stress [3,4,5]. Some mechanisms of action have been proposed for its anxiolytic and antidepressant activity. Lopez et al. [5] reported that lavender oil, linalyl acetate and linalool (the main constituents from lavender oil) act as antagonists on glutamate NMDA-receptor (N-Methyl-d-aspartate receptor). They have also shown that lavender oil and linalool bind to the serotonin transporter (SERT); hence they could modulate the serotoninergic transmission. Its anxiolytic and antidepressant effects have also been researched in clinical trials and the results were promising. In some cases, the effects were similar to that of SSRI’s (Selective serotonin reuptake inhibitors) [6].

The pharmacological actions of lavender oil are strictly linked with its composition. According to European Pharmacopoeia 8th Ed [7], the chemical compounds should be between the following limits: linalyl acetate: 25–47%, linalool: 20–45%, terpinen-4-ol: 0.1–8%, 3-octanone: 0.1–5%, 1,8-cineole: max. 2.5%, α-terpineol: max. 2%, camphor: max. 1.2%, limonene: max. 1%, lavandulyl acetate: min. 0.2%, lavandulol: min. 0.1%. The concentration of essential oil in the dried herbal drug should be min 1.3%.

The cultivation conditions (type of soil, climatic conditions and fertilizers) have an extremely important role in the concentration of the phytoconstituents in plants. Biostimulants are natural products that, applied in low quantities, promote plant growth [8].

The rhytidome (bark) is a set of multiple layers of periderms, with protective role for woody vascular plants against overheating, frost, herbivores or infestation with parasites [9]. The bark (20% of the dry weight of woody vascular plants) contains lignin, polysaccharides, suberin, phenolic compounds [9]. The spruce (*Picea abies* L.) and beech (*Fagus sylvatica* L.) are some of the most widespread woody vascular plants in Europe and particularly in Romania, with a high economic value [10]. Spruce and beech wood is mostly used in the wood processing industry or for fire wood. After processing, a significant amount of bark is obtained. Thus, spruce and beech bark are considered a waste product in the wood industry [11]. Our previous results have shown that beech and spruce bark crude extract has antimicrobial, antitumoral and bioregulator effect in sage plants [10,11,12]. Due to their high polyphenolic content, their utilization as biostimulants could have an important ecological impact, and this process could be further exploited in the production of organic essential oils.

The aim of this paper is to assess the influence of spruce and beech bark crude extracts on the growth and development (germination capacity, biomass production, histo-anatomical aspects, photo-assimilatory pigment accumulation) of lavender plants, with special attention to quantitative and qualitative analysis of volatile oil obtained from the lavender flowers and leaves.

## 2. Materials and Methods

### 2.1. Plant Sample and Chemicals

Bark (rhytidome) is a set of dead tissues (multiple layers of periderms), which forms the protective layers of woody vascular plants. Spruce (*Picea abies* L.) and beech (*Fagus sylvatica* L.) bark was provided from the forest of the Gurghiului Mountains, Mureș County, Romania, during November and December 2017. The trees were about 15–20 years old. The bark was collected and splintered manually from the stems of the beech and spruce trees. The plants were identified and authenticated by Dr. Corneliu Tanase. The bark was air-dried (10.5% humidity, room temperature) and milled in a GRINDOMIX GM 2000 mill to a mean particle size diameter of < 0.5 mm. Immediately after grinding the extraction process followed. The bark was used without any pre-treatments.

The lavender (*Lavandula angustifolia* Mill.) seeds come from the seeds collection of the Botanical Garden of the University of Medicine, Pharmacy, Sciences and Technology “G.E. Palade” from Târgu Mureș, being collected in 2017.

All chemicals and standards were provided by Sigma-Aldrich (St. Louis, MO, USA).

### 2.2. Extraction

The aqueous extracts were obtained by applying a classical batch water extraction, using 10 g of the grounded and dried bark and 300 mL of distilled water. The mixture was kept in a water bath (45 min, 85 to 90 °C). The extraction was repeated three times. The bark extracts were filtered and put together in a 1000 mL volumetric flask and completed to volume with distilled water. The extract concentration obtained was 1000 mg/100 mL. The extracts were diluted with tap water 1:1, obtaining a concentration of 500 mg/100 mL and freshly used.

The tested solutions (experimental variants) were as follows: tap water (Control), spruce bark extract at 500 mg dry bark/100 mL solvent (SBE500), spruce bark extract at 1000 mg dry bark/100 mL solvent (SBE1000), beech bark extract at 500 mg dry bark/100 mL solvent (BBE500), beech bark extract at 1000 mg dry bark/100 mL solvent (BBE1000).

Extraction of the volatile oil from lavender plants was performed by hydro-distillation, using a Neo-Clevenger device (1000 mL water/10 g of plant material, moderate distillation speed). The essential oil content was expressed in mL/100 g on the dry weight basis (DW), according to the ISO 3515:2002 standard for lavender oil and European Pharmacopoeia [7]. The obtained essential oil was dried on anhydrous sodium sulphate and was stored at 4 °C in the refrigerator, in amber bottles, until analysis.

### 2.3. Working Protocol

The experiment was conducted in the Botanical Garden of “George Emil Palade” University of Medicine, Pharmacy, Sciences and Technology from Târgu Mureș, through the following steps:

- the lavender (*Lavandula angustifolia* Mill.) seeds were sterilized (immersion in a 20% HClO solution for 2 min and well washed with water).
- the seeds were carefully selected and then immersed (first application) in the tested extracts (BBE500, BBE1000, SBE500, SBE1000) for 12 h, at a constant temperature of 25 °C.
- the seeds (3 seeds/pot and 3x60 seeds/experimental variant) were sown manually into pots (60 pots/experimental variant).
- the pots were wetted with 20 mL of tested extracts/pot (second application).
- at vegetative stage, after 30 days from germination, the plants were wetted with 20 mL/of tested extracts/pot (third application—at radicular level).
- after 60 days from the beginning of the experiments, the lavender plants were transferred in the field.
- after 1 year, at vegetative stage, the plants were wetted with 10 mL of tested extracts/plant (fourth application—at foliar level by spraying).
- during the flowering stage, the aerial part of the plant was harvested, separating the plant organs into inflorescences (flos) and stems with leaves (herba). The plant material was dried in the open air, being prepared for hydrodistillation.

### 2.4. Plant Growth and Development Analysis

The germination capacity was recorded when the radicle was at least 2 mm long. Germination capacity (CG) was calculated as follows: CG (%) = (Total number of seeds germinated/Total number of seeds tested) × 100 [13].

To evaluate the influence of bark extracts on lavender growth and development, the plants were separated into inflorescences and stems with leaves. The biomass determination was done by gravimetric methods.

The UV-Vis absorption spectra were acquired by using a UV-Vis spectrophotometer (Specord, 2100), at room temperature, by using quartz cells. For pigment quantification [14], 0.05 g of fresh vegetal material (collected at vegetative stage) was milled with quartz sand and extracted with acetone (80%). The experiment was performed in triplicate. The chlorophyll a and b, and carotenoid contents were determined at wavelengths of 470, 646, and 663 nm and quantified in accord with Lichtenthaler and Wellburn [14]:

Chlorophyll a (µg/mL) = 12.21*A663 − 2.81*A646(1)

Chlorophyll b (µg/mL) = 20.31*A646 − 5.03*A663(2)

Carotenes (µg/mL) = (100*A470 − 3.27*[chl a] − 104*[chl b])/22(3)

### 2.5. Histo-Anatomical Analysis

The cross-sections of the stem and leaves were double-stained using iodine green and ruthenium red [15] and analyzed with a Motic Microscope and photographed with a Nikon Coolpix L22 camera, Tokyo, Japan. The image analysis was performed with ImageJ Image Processing and Analysis in Java Version 1.51j8 (National Institute of Mental Health, Bethesda, MD, United States). For each experimental variant, 25 samples/vegetative organs were analyzed.

### 2.6. GC-MS Qualitative Analysis of Volatile Compounds

ITEX/GC-MS

The extraction of volatiles from lavender flos and folium was performed using the ITEX (in-tube extraction) technique [16] followed by their separation and identification by gas-chromatography—mass spectrometry (GC-MS), using a GC-MS Shimadzu model QP-2010 (Shimadzu Scientific Instruments, Kyoto, Japan) equipped with a Combi-PAL AOC-5000 autosampler (CTC Analytics, Zwingen, Switzerland) and a capillary column (ZB-5 ms, 30 m × 0.25 mm i.d. × 0.25 µm, Phenomenex, Torrance, USA).

Shortly, for the extraction of the volatiles, 0.15 g of sample was introduced in a headspace vial (20 mL), hermetically closed and incubated at 60 °C under continuous agitation (500 rpm) for 10 min. After incubation, the needle of the headspace syringe was introduced into the headspace of the vial and using the syringe plunger, the volatile compounds were adsorbed repeatedly (15 strokes) into a porous polymer fiber microtrap (ITEX-2TRAPTXTA, Tenax TA 80/100 mesh, ea) placed between the syringe needle and body. The extraction speed was set at 100 μL/s, a volume of 1000 μL from the headspace phase being adsorbed with each stroke. The thermal desorption of volatiles was made directly into the GC–MS injector, after which the hot trap (250 °C) was cleaned with N_2_. All of the above operations were perform automatically using the equipment autosampler (Combi-PAL AOC-5000 autosampler, CTC Analytics, Zwingen, Switzerland).

The separation of volatile compounds on the ZB-5ms capillary column was performed using the method described by Tanase et al., 2020 [10]. The column temperature program started from 50 °C (held for 2 min) and increased to 160 °C with a rate of 4°/min and then to 250 °C with a 15°/min rate and held at 250 °C for 10 min. The flow rate of the carrier gas (helium) was set at 1 mL/min. The temperature for the injector, ionic source and interface were set at 250 °C. The MS detection was performed on a quadrupole mass spectrometer operating in full scan (40–500 m/z), with electron impact (EI) as ion source at an ionization energy of 70 eV. The tentative identification of the volatile constituents was achieved by matching their recorded mass spectra and retention times with those of standard compounds (β–myrcene, limonene, α-pinene, β–pinene, caryophyllene, β– cis-farnesene, geraniol, nerol, caryophyllene oxide), using the software’s NIST27 and NIST147 mass spectra libraries (considering a minimum similarity of 85%) and by comparison of the fragmentation patterns of the mass spectra with those from databases and verified with retention indices drawn from www.pherobase.com or www.flavornet.org for columns with a similar stationary phase. Thus, a qualitative assessment of volatile compounds was achieved, the relative percentage of each compound being estimated as a fraction of its integrated ion area from the total ion chromatograms (TIC) area (100%).

### 2.7. Statistical Analysis

The experiment was performed in triplicate. The statistical tests used were the Kruskal-Wallis test for including a sample in a specific distribution and Mann-Whitney U test (non-parametric) for comparing two population means. The measurements were normally distributed. The tests were applied in Past 2.17. One-way ANOVA, followed by Tukey Kramer posthoc test, was used to compare the differences between groups for the chemical compounds quantified in the essential oil. The data values were defined to be statistically significant at *p* < 0.05.

## 3. Results

### 3.1. Seed Germination

The data showed that the tested solutions have a stimulatory influence on the lavender seed’s germination capacity compared to the control (Table 1). A significant statistic germination stimulating effect was recorded for BBE1000 (with 34% higher compared with the control) and for SBE500 (with 17% higher compared with control). For the SBE1000 and BBE500 the stimulation percentage, compared to the control, was statistically insignificant.

### 3.2. Biomass Accumulation

**Figure 1:**
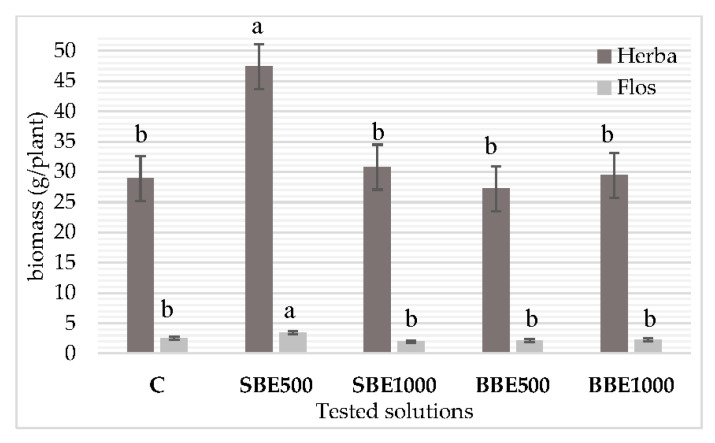
The influence of bark extracts on *Lavandula angustifolia* Mill. biomass accumulation. Error bars represent the standard deviations of means. C—Control, SBE500—spruce bark extract at 500 mg dry bark/100 mL solvent, SBE1000—spruce bark extract at 1000 mg dry bark/100 mL solvent, BBE500—beech bark extract at 500 mg dry bark/100 mL solvent, BBE1000—beech bark extract at 1000 mg dry bark/100 mL solvent. Different letters (a, b) for the same color mean statistically significant differences at *p* < 0.05.

There were no significant differences in biomass production between SBE100, BBE500 and BBE1000 and control, as observed in Figure 1. However, the amount of herba and flos biomass was significantly (*p* ≤ 0.05) higher at SBE500 compared to the control. Thus, at a lower concentration (0.5 g spruce bark/100 mL extract) the stimulation percentage of SBE500 was 64% for herba and 32% for flos.

### 3.3. Photo-Assimilating Pigment Content in Lavender Leaves

An increase in photo-assimilating pigments (chlorophyll a, chlorophyll b and carotenes) was noticed for all experimental variants compared to control (Table 2). The stimulating effect on the pigments production by beech and spruce extracts was highlighted especially in the chlorophyll a content of where statistically significant differences for SBE500, BBE500 and BBE1000 variants were noticed.

### 3.4. Histo-Anatomical Aspects of the Lavender Stem and Leaves

**Figure 2:**
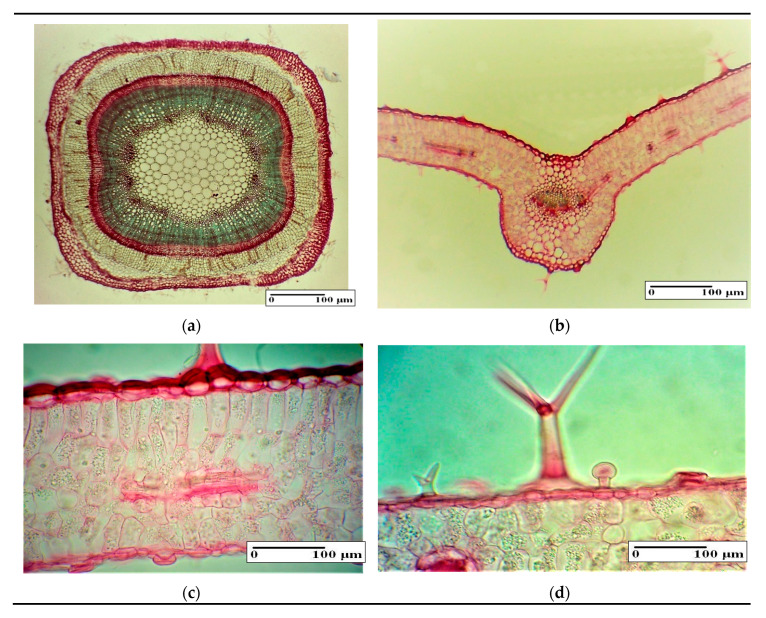
The general aspects of internal structure of *Lavandula angustifolia* Mill. vegetative organs: stem (**a**), main string of leaf (**b**), leaf lamina (**c**) and leaf lamina with protective and glandular trichomes (**d**).

The stem cross-section contour is quadratic with angular collenchym, present in the four ribs (Figure 2a). The epidermis has isodiametric cells and shows protective and glandular trichomes. The cortical tissue is well developed. The central cylinder contains the vascular tissues. In the section center is cellulose parenchymatic pith. Following the measurements and the statistical analysis, no significant differences were observed between the experimental variants and the control (Table 3).

The equifacial structure of the leaf (Figure 2b) present two epidermis with single layer and mesophyll with palisade parenchyma on both sides (Figure 2c). On both leaf surfaces there are protective and glandular trichomes (Figure 2d). The microscopic measurements at the leaf level showed statistically significant differences between SBE500 and control (Table 3). Thus, the leaf lamina/mesophyll thickness at SBE500 is significantly larger compared to the control, showing a better development of the leaf tissue. These results correlate with the higher amount of foliar biomass in the SBE500 variant, shown in this work previously.

### 3.5. Volatile oil Content Analysis

#### 3.5.1. Quantitative Analysis of Volatile oil from Lavandulae Flos

**Figure 3:**
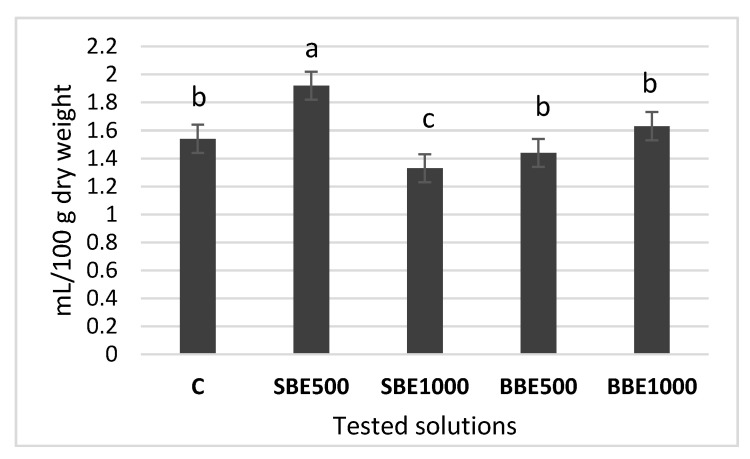
Volatile oil content from Lavandulae flos (mL/100 g dry weight); C—Control, SBE500—spruce bark extract at 500 mg dry bark/100 mL solvent, SBE1000—spruce bark extract at 1000 mg dry bark/100 mL solvent, BBE500—beech bark extract at 500 mg dry bark/100 mL solvent, BBE1000—beech bark extract at 1000 mg dry bark/100 mL solvent. Different letters (a, b, c) mean statistical significant differences at *p* < 0.05.

According to the literature, fresh lavender flowers contain about 0.8% and those dried up to 1.5% volatile oil, being a mixture of over 100 volatile compounds of which linalool and linalyl acetate dominate quantitatively. The lavender oil is colorless or slightly yellow, with an aromatic odor and a slightly bitter taste. Following the hydrodistillation, values between 1.33–1.92% were recorded (Figure 3). Significant differences, compared to the control, appear only in the SBE500 variant, where the highest amount of volatile oil was obtained, namely 1.92 mL/100g flos. Thus, the results regarding the quantity of volatile oil are correlated positively with those for the lavender flowers biomass production.

#### 3.5.2. ITEX/GC-MS Analysis of Volatile Compounds from Lavandulae Flos

**Figure 4:**
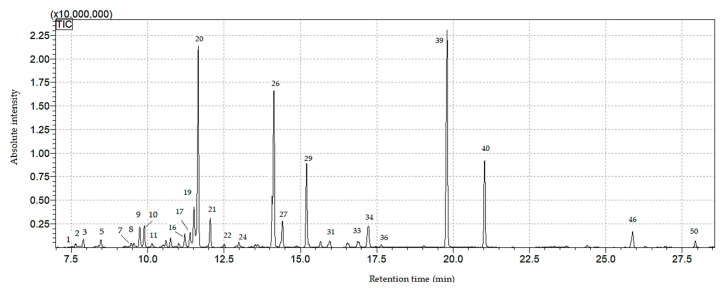
ITEX/GC-MS chromatogram of the volatile compounds from Lavandulae flos (SBE1000) sample (for peak numbering see Table 4).

Forty-five volatile compounds were found (present by means of the commercial spectral libraries) from Lavandulae flos (Figure 4 and Table 4). The most quantitatively predominant volatile compounds were: bergamiol, β—linalool, lavandulol, β—ocimene, D-limonene, allo-ocimene. The compounds separated and identified from Lavandulae flos are presented in Table 4 and are expressed as percentages of total peak area. Some volatile compounds were identified only in experimental variants. Thus, cyclen was identified in SBE1000 and BBE1000, borneol in SBE1000, BBE500 and BBE1000, cryptone, santalen and caryophyllene oxide only in BBE500 and β—Farnesene in SBE500, SBE1000 and BBE1000. One the other hand, α- Phellandrene and cis-Sabinenhydrate was identified, at a low percentage, only in control.

Analyzing the percentage of main volatile compounds in the experimental variants, it was found that there were differences compared to the control. Thus, D—Limonene and eucalyptol concentration was higher in BBE500 (13.31% respectively 8.18%) compared with control (2.99%, respectively 2.76%), but β—Ocimene concentration decreased in BBE500 (8.87%) compared to control (19.04%). The higher concentration of bergamiol was in BBE1000 (33.39%), comparing with control (24.02%). The lavandulol concentration was higher in SBE1000 and BBE500 experimental variants.

#### 3.5.3. ITEX/GC-MS Analysis of Volatile Compound from Lavandulae Folium

Twenty-seven volatile compounds were found (present by means of the commercial spectral libraries) from Lavandulae folium (Table 5). The most quantitatively predominant volatile compounds in Lavandulae folium were: eucalyptol, p-Cymene, D-Limonene and camphor. The compounds separated and identified from Lavandulae folium (Table 5) are expressed as percentages of total peak area.

From Table 5, it is observed that in Control were a few identified compounds (16 compounds) comparing with experimental variants (SBE500—24 compounds, SBE1000—31 compounds, BBE500—17 compounds, BBE1000—29 compounds). The main compounds identified only in experimental variants were α- Thujene, sabinene, β—Myrcene, 3—Carene and α- santalene.

Analyzing the concentration of volatile compounds in the experimental variants, there were differences compared to the control. For example, the percentage of camphene in SBE500 was 11.15%, higher than the control, where it is 3.09%. D-Limonene is in higher concentration for experimental variants (25.64%—SBE1000, 27.15%—BBE500, 32.76%—BBE1000) comparing with control (12.54%) but eucalyptol concentration decreases for SBE1000 and BBE500 (3.56%—SBE1000, 11.98%—BBE500), compared with control (25.12%). The borneol concentration was higher in BBE500 (13.9%) comparing with control (9.6%) and with BBE1000 (1.4%).

## 4. Discussions

The beech and spruce aqueous bark extracts was characterized previously regarding total polyphenol and tannins content [11]. Phenolic compounds were identified in these extracts, such as gallic acid, vanillic acid, epicatechin, catechin, protocatechuic acid, chlorogenic acid, ferulic acid, quercetin and isoquercitrin [12,17]. Some of these compounds could have an important bioregulator role in lavender plant growth and development. For example, it has been shown that vanillic acid stimulates the growth of maize seedlings [18].

The obtained results show the rich effect of the beech bark extract on the germination process. The stimulatory effect of beech bark extract on sage germination and biomass production was observed previously [10]. The results suggest possible enzymatic changes induced by BBE1000, influencing the lavender seeds germination capacity [19]. The germination process of lavender seeds is generally possible after breaking seed dormancy by freezing or using plant hormones [20]. Because there was no other seeds pretreatment, the results obtained are promising for the enhancement of germination capacity. On the other hand, the spruce extract caused an increase in the leaves biomass and an increase in the amount of photoassimilatory pigments synthesized. Considering previous results [21], it was concluded that global extracts induce similar effects of auxins and cytokinins in plant growth. The stimulating effect was most often obtained at a 0.5 g/L global extract concentration. At a concentration greater than 1 g/L, an inhibitory effect is observed [21].

For lavender growers, the main objective is to obtain plants with higher essential oil content while preserving the quality of the essential oil. The essential oil obtained from *Lavandula angustifolia* is considered to have the highest quality, but with the disadvantage that the yield is usually lower than in other *Lavandula* species [22].

In our previous research, we have shown that bark extracts act as bioregulators in sage crops, influencing the production of secondary metabolites [10]. Plant polyphenols have been reported to act as stimulators in metabolic processes in plants, with positive effects on the final product quality. Still, the mechanism by which these compounds affect the metabolic pathway is not entirely understood. Some authors suggest that plant polyphenols increase the activity of some enzymes by modulating carbon and nitrogen metabolism [23]. However, recent data suggest that the influence of biostimulators has a dose-dependent manner, and the studies have demonstrated the existence of a maximum dose, which, once overcome, inhibits plant metabolism [24]. Our findings are in accordance with these studies.

In the present paper, the essential oil yield was significantly higher (*p* < 0.05) in the SBE500 variety while in SBE1000 it was significantly lower (*p* < 0.05) compared to control. No differences were noticed in the BBE500 and BBE1000 varieties.

According to the European Pharmacopoeia, lavender essential oil should contain a maximum concentration of eucalyptol (1,8 cineole) of 2.5%, therefore only the essential oil from SBE500 variety fits within these limits. The ISO 3515:2002 standard for lavender oil provides the limits for the most important compounds: linalool (25–38%), linalyl acetate (25–45%) and camphor (0.5–1.0%). Limonene concentration should be lower than 1% according to Eur. Ph. and lower than 0.5% according to the ISO standards, probably because of the D-limonene sensitization properties [25]. All tested varieties had a significant lower limonene content than control (*p* < 0.05).

The essential oil obtained from BBE1000 variety had the highest content of linalyl acetate of all the tested samples and is the only one that meets the ISO aforementioned standards for linalyl acetate. Regarding the linalool content, the oil obtained from the SBE500 variety is between the limits of the ISO standard. Usually, the quality criteria of lavender essential oil are based on the concentration of linalool and linalyl acetate, and these should be present in almost equal quantities [26]. Alpha and beta-pinene were in significant higher concentrations in the BBE500 variety compared to control. These compounds have been shown to possess neuroprotective effects by regulating the cognitive processes [27,28]. They also have remarkable antimicrobial activity against Gram-positive and Gram-negative strains and also against fungi [28,29,30],

Regarding the chemical composition of essential oil obtained from the leaves, significant differences were noticed in the four variants compared to control. In the essential oil obtained from the BBE1000 variety, significantly lower concentrations were noticed, especially for the oxygenated compounds, while the hydrocarbons were in higher levels. Although some authors suggest that the main compounds found in lavender leaves are 1,8-cineole and camphor, in our samples the dominant compounds were found to be p-cymene and 1,8-cineole.

In conclusions, this preliminary results, showed that the bark phenolic extract can have biological activities and influence the production of volatile oil in *Lavandula angustifolia* and also interferes somewhere in the monoterpene pathway, causing significant changes in the phytochemical profile of the essential oil.

The obtained results suggest future prospects with promising bark extract treatments and concentrations with the degree of enhancement of different growing and volatile parameters of lavender or other aromatic plants. Future research is needed to identify which compounds from bark extracts influence the growth, development, and chemical profile of lavender plants or essential oil content. Extensive studies are needed to see if these effects occur due to the action of a single phenolic compound, or whether the total polyphenols have a synergistic effect.

## Figures and Tables

**Table 1:** The influence of bark extracts on lavender seed germination capacity.

Tested Solutions	Total Seeds Number	GC (%)	SD (±)	SCC (%)
C	198	60.10 ^b^	7.00	-
SBE500	198	70.20 ^a^	6.31	16.81
SBE1000	198	64.65 ^b^	7.29	7.56
BBE500	198	66.16 ^b^	7.32	10.08
BBE1000	198	80.81 ^a^	6.25	34.45

C—Control, SBE500—spruce bark extract at 500 mg dry bark/100 mL solvent, SBE1000—spruce bark extract at 1000 mg dry bark/100 mL solvent, BBE500—beech bark extract at 500 mg dry bark/100 mL solvent, BBE1000—beech bark extract at 1000 mg dry bark/100 mL solvent. GC—germination capacity. SCC—Stimulation compared to control. Different superscript letters (a, b) in the same column mean statistically significant differences at *p* < 0.05.

**Table 2:** Content of photo-assimilating pigments synthesized (µg/g) in lavender leaves.

Experimental Variant	Chl a	Chl b	Chl a + Chl b	Chl a/Chl b	Carotens
C	64.85 ± 5.08 ^b^	11.71 ± 1.82	76.57	5.54	2.04 ± 0.01
SBE500	102.86 ± 8.00 ^a^	19.44 ± 1.09	122.29	5.29	2.83 ± 0.02
SBE1000	84.66 ± 7.57 ^b^	15.11 ± 1.09	99.77	5.6	2.54 ± 0.02
BBE500	101.21 ± 6.87 ^a^	20.41 ± 1.09	121.62	4.96	2.48 ± 0.02
BBE1000	97.22 ± 11.06 ^a^	19.53 ± 2.03	116.74	4.98	2.56 ± 0.05

C—Control, SBE500—spruce bark extract at 500 mg dry bark/100 mL solvent, SBE1000—spruce bark extract at 1000 mg dry bark/100 mL solvent, BBE500—beech bark extract at 500 mg dry bark/100 mL solvent, BBE1000—beech bark extract at 1000 mg dry bark/100 mL solvent. Chl a—chlorophyll a; Chl b—chlorophyll b. Different superscript letters (a, b) in the same column mean statistically significant differences at *p* < 0.05.

**Table 3:** The microscopic characteristics of vegetative organs in case of treated and control lavender plants.

Vegetative Organs	Microscopic Characteristics	Control (Mean ± SD)	Experimental Variants (Mean ± SD)			
			SBE500 ^1^	SBE1000^1^	BBE500 ^1^	BBE1000 ^1^
		n = 25	n = 25	n = 25	n = 25	n = 25
Stem	Epiderm and cortex area (%)	37.71 ± 2.41 ^ab^	38.89 ± 2.16 ^a^	36.27 ± 2.14 ^bc^	35.05 ± 1.14 ^c^	36.07 ± 1.87 ^c^
	Floem area (%)	13.85 ± 1.49 ^a^	14.11 ± 1.25^a^	13.35 ± 1.31 ^ab^	12.35 ± 1.51 ^b^	13.37 ± 1.22 ^ab^
	Xylem area (%)	28.99 ± 1.97 ^ab^	27.46 ± 2.77 ^a^	30.28 ± 1.87 ^bc^	29.87 ± 1.98 ^bc^	30.91 ± 2.01 ^c^
	Pith area (%)	15.87 ± 1.29 ^a^	15.82 ± 1.43 ^a^	16.38 ± 2.17 ^a^	18.44 ± 2.37 ^b^	16.16 ± 1.37 ^a^
	Colenchim area (%)	3.58 ± 0.87 ^a^	3.72 ± 0.84 ^ab^	3.09± 0.91 ^a^	4.29 ± 0.83 ^b^	3.49 ± 0.82 ^a^
Leaf	Leaf lamina thickness (mm)	0.052 ± 0.006 ^b^	0.068 ± 0.009 ^a^	0.054 ± 0.004 ^b^	0.054 ± 0.005 ^b^	0.053 ± 0.005 ^b^
	Mesophyll thickness (mm)	0.043 ± 0.004 ^b^	0.059 ± 0.006 ^a^	0.041 ± 0.004 ^b^	0.041 ± 0.004 ^b^	0.044 ± 0.004 ^b^
	Vascular bundles area in the main string (%)	18.55 ± 1.44 ^a^	20.42 ± 2.03 ^b^	18.02 ± 2.11 ^a^	17.11 ± 3.02 ^a^	18.75 ± 2.02 ^a^

^1^ C—Control, SBE500—spruce bark extract at 500 mg dry bark/100 mL solvent, SBE1000—spruce bark extract at 1000 mg dry bark/100 mL solvent, BBE500—beech bark extract at 500 mg dry bark/100 mL solvent, BBE1000—beech bark extract at 1000 mg dry bark/100 mL solvent; ± SD (standard deviation). Different superscript letters (a, b, c) in the same column mean statistically significant differences at *p* < 0.05.

**Table 4:** Volatile oil composition of lavender flowers.

No	Compounds	Retention Time	Concentration (% of Total Surface Area of Peaks)				
			C ^1^	SBE500 ^1^	SBE1000 ^1^	BBE500 ^1^	BBE1000 ^1^
1	Cyclene	7.522	-	-	0.06 ± 0.01	-	0.13 ± 0.05
2	α- Thujene	7.634	0.5 ± 0.05	0.25 ± 0.04	0.23 ± 0.05	0.4 ± 0.02	0.21 ± 0.04
3	α- Pinene	7.889	0.71 ± 0.06 ^a^	0.43 ± 0.05 ^a^	0.62 ± 0.03 ^a^	1.91 ± 0.11 ^b^	0.43 ± 0.03 ^a^
4	Dimethylcrotonolactone*	8.407	0.09 ± 0.02				
5	Camphene	8.467	0.11 ± 0.03 ^a^	0.13 ± 0.02 ^a^	0.69 ± 0.06 ^abc^	0.41 ± 0.05 ^b^	0.83 ± 0.05 ^c^
6	Sabinen	9.288	0.18 ± 0.02			0.49 ± 0.03	
7	β—Pinene	9.459	0.43 ± 0.02 ^a^	0.24 ± 0.02 ^a^	0.29 ± 0.02 ^a^	3.07 ± 0.08 ^b^	0.17 ± 0.02 ^a^
8	1-Octen-3-ol	9.547	0.39 ± 0.02	0.58 ± 0.07	0.27 ± 0.03	0.33 ± 0.03	0.21 ± 0.03
9	3-Octanone	9.739	1.29 ± 0.11 ^a^	2.82 ± 0.09 ^b^	1.73 ± 0.08 ^b^	0.86 ± 0.03 ^d^	5.36 ± 0.12 ^e^
10	β—Myrcene	9.885	2.15 ± 0.08 ^ab^	2.24 ± 0.05 ^a^	1.83 ± 0.06 ^bc^	1.59 ± 0.06 ^c^	2.72 ± 0.09 ^d^
11	Butanoic acid	10.137	0.25 ± 0.04	0.29 ± 0.03	0.33 ± 0.05	0.37 ± 0.03	0.19 ± 0.02
12	α- Phellandrene	10.517	0.29 ± 0.01				0.14 ± 0.01
13	3-Carene*	10.596	0.66 ± 0.07	0.6 ± 0.09	0.57 ± 0.08	0.35 ± 0.02	0.49 ± 0.07
14	Acetic acid	10.748	0.06 ± 0.01 ^a^	1.26 ± 0.11 ^d^	0.74 ± 0.06 ^b^	0.32 ± 0.05 ^ac^	0.49 ± 0.05 ^bc^
15	4-Carene*	11.018			0.23 ± 0.02		
16	p-Cymene	11.22	1.24 ± 0.05 ^a^	1.15 ± 0.06 ^a^	1.03 ± 0.07 ^a^	6.28 ± 0.12 ^b^	1.09 ± 0.04 ^a^
17	D-Limonene	11.386	2.99 ± 0.07 ^a^	2.05 ± 0.05 ^c^	1.32 ± 0.03 ^b^	1.33 ± 0.04 ^b^	0.84 ± 0.02^b^
18	β- Phellandrene	11.453	0.81 ± 0.07				
19	Eucalyptol	11.52	2.76 ± 0.09 ^a^	1.22 ± 0.07 ^b^	4.53 ± 0.10 ^c^	8.18 ± 0.11 ^d^	0
20	β—trans-Ocimene	11.647	11.14 ± 0.37 ^a^	16.42 ± 0.44 ^b^	18.17 ± 0.54 ^c^	7.29 ± 0.44 ^d^	15.21 ± 0.23 ^e^
21	β—cis-Ocimene	12.047	7.9 ± 0.15 ^a^	3.88 ± 0.09 ^b^	2.58 ± 0.11 ^c^	1.68 ± 0.08 ^d^	8.82 ± 0.27 ^e^
22	γ—Terpinene	12.501	0.34 ± 0.04	0.19 ± 0.03	0.26 ± 0.07	0.11 ± 0.03	0.19 ± 0.04
23	cis-Sabinenhydrate	12.897	0.21 ± 0.07				
24	cis-Linalool oxide	12.99	0.37 ± 0.06 ^a^	0.62 ± 0.07 ^a^	0.47 ± 0.05 ^a^	2.79 ± 0.09 ^b^	
25	1,2-Oxolinalool	13.61	0.16 ± 0.06 ^a^	0.38 ± 0.07 ^a^	0.27 ± 0.04 ^a^	1.92 ± 0.11 ^b^	
26	β—Linalool	14.127	20.02 ± 0.45 ^a^	26.98 ± 0.76 ^b^	14.75 ± 0.32 ^c^	17.43 ± 0.48 ^d^	15.34 ± 0.51 ^e^
27	1-Octenyl acetate	14.407	0.65 ± 0.03 ^a^	0.87 ± 0.04 ^a^	2.35 ± 0.08 ^b^	0.56 ± 0.07 ^a^	1.45 ± 0.12 ^b^
28	3-Octyl acetate	14.87	0.08 ± 0.02	0.25 ± 0.04	0.12 ± 0.04		0.25 ± 0.03
29	allo-Ocimene	15.195	5.59 ± 0.44 ^a^	6.37 ± 0.22 ^b^	7.88 ± 0.15 ^c^	2.33 ± 0.04 ^d^	5.45 ± 0.11 ^a^
30	n.i.	15.655			0.59 ± 0.21		
31	Camphor	15.96		0.19 ± 0.03 ^b^	0.84 ± 0.04 ^a^	0.54 ± 0.07 ^b^	0.32 ± 0.05 ^ab^
32	Lavandulol	16.563	0.94 ± 0.06	1.00 ± 0.12		1.24 ± 0.14	
33	Borneol	16.9			0.85 ± 0.13 ^a^	0.66 ± 0.07 ^b^	0.4 ± 0.11 ^ab^
34	1-Terpinen-4-ol	17.221	4.66 ± 0.12 ^a^	2.06 ± 0.11 ^b^	1.71 ± 0.08 ^bc^	1.42 ± 0.11 ^c^	1.74 ± 0.09 ^bc^
35	Cryptone					1.08 ± 0.09	
36	Butyric acid	17.65	0.18 ± 0.05	0.46 ± 0.07	0.27 ± 0.02	0.44 ± 0.12	0.28 ± 0.05
37	n.i.	18.601				0.14 ± 0.03	
38	Isoborneol	19.041			0.18 ± 0.02		
39	Linalyl acetate	19.803	24.02 ± 0.27 ^a^	18.52 ± 0.42 ^b^	22.44 ± 0.54 ^c^	11.35 ± 0.88 ^d^	33.39 ± 1.26e
40	Lavandulyl Acetate	21.03	5.05 ± 0.61 ^a^	5.19 ± 0.07 ^a^	8.65 ± 0.97 ^b^	8.21 ± 0.74 ^b^	1.28 ± 0.06 ^b^
41	n.i.	23.202				0.25 ± 0.06	
42	n.i.	23.3				0.27 ± 0.05	
43	trans-Geraniol*	23.71	0.15 ± 0.02	0.21 ± 0.02	0.12 ± 0.03	0.18 ± 0.06	0.21 ± 0.04
44	cis-Geraniol	24.391	0.31 ± 0.09	0.36 ± 0.02	0.27 ± 0.03	0.39 ± 0.05	0.27 ± 0.05
45	Santalen	25.839				1.46 ± 0.07	
46	Caryophyllene	25.868	1.62 ± 0.10 ^a^	1.81 ± 0.08 ^ab^	2.12 ± 0.07 ^b^		1.55 ± 0.10 ^a^
47	α—trans-Bergamotene	26.299	0.13 ± 0.08	0.06 ± 0.01	0.05 ± 0.01		0.12 ± 0.03
48	β – cis-Farnesene *	26.928		0.33 ± 0.10	0.13 ± 0.05		0.32 ± 0.06
49	n.i.	27.922	0.37 ± 0.08				0.12
50	n.i.	27.931		0.59 ± 0.06	0.46 ± 0.07		
51	Caryophyllene oxide	30.894				0.37 ± 0.05	
	Total % of identified compounds		99.63	99.41	98.95	99.34	99.88

^1^ C—Control, SBE500—spruce bark extract at 500 mg dry bark/100 mL solvent, SBE1000—spruce bark extract at 1000 mg dry bark/100 mL solvent, BBE500—beech bark extract at 500 mg dry bark/100 mL solvent, BBE1000—beech bark extract at 1000 mg dry bark/100 mL solvent; n.i.—not identified, *—tentative identification. Different letters in the same row mean statistically significant differences at *p* < 0.05.

**Table 5:** Voil composition of lavender leaves.

Compounds	Retention Time	Concentration (% of Total Surface Area of Peaks)				
		C ^1^	SBE500 ^1^	SBE1000 ^1^	BBE500 ^1^	BBE1000 ^1^
Tricyclene	7.523		0.79 ± 0.05	0.22 ± 0.05		0.27 ± 0.03
α- Thujene	7.638		0.82 ± 0.02	0.95 ± 0.08	1.09 ± 0.11	0.92 ± 0.08
α- Pinene	7.891	1.97 ± 0.07 ^a^	4.9 ± 0.25 ^b^	4.17 ± 0.46 ^c^	3.19 ± 0.54 ^d^	1.82 ± 0.11 ^a^
Camphene	8.472	3.09 ± 0.51 ^ac^	11.15 ± 1.07 ^b^	3.49 ± 0.24 ^a^	3.04 ± 0.44 ^ac^	2.73 ± 0.28 ^c^
Sabinene	9.223		1.98 ± 0.5 ^ab^	3.2 ± 0.33 ^a^	1.34 ± 0.03 ^b^	1.42 ± 0.41 ^b^
Β—Terpinene *	9.276				1.33 ± 0.51	1.12 ± 0.11
β—Pinene	9.463	2.99 ± 0.11 ^ab^	2.45 ± 0.12 ^b^	3.17 ± 0.08 ^ac^	3.13 ± 0.09 ^b^	1.09 ± 0.06 ^c^
1-Octen-3-ol	9.544	2.26 ± 0.11 ^a^	0.5 ± 0.04 ^b^	1.92 ± 0.07 ^a^		0.83 ± 0.04 ^b^
3-Octanone	9.743	2.09 ± 0.12 ^a^	0.32 ± 0.11 ^b^	0.71 ± 0.08 ^b^		1.07 ± 0.04 ^c^
β—Myrcene	9.894		0.46 ± 0.07 ^a^	0.28 ± 0.03 ^a^	1.41 ± 0.05 ^b^	0.83 ± 0.06 ^ab^
3-Carene*	10.594		5.79 ± 0.12 ^a^	4.23 ± 0.22 ^b^	5.96 ± 0.23 ^a^	2.97 ± 0.31 ^b^
p-Cymene	11.213	24.95 ± 1.22 ^a^	19.35 ± 1.08 ^b^	24.94 ± 1.05 ^a^	15.84 ± 1.13 ^c^	17.03 ± 1.27 ^d^
D-Limonene	11.386	12.54 ± 0.97 ^a^	12.49 ± 0.86 ^a^	25.64 ± 1.02 ^b^	27.15 ± 1.11 ^c^	32.76 ± 0.98 ^d^
Eucalyptol	11.508	25.12 ± 0.75 ^a^	20.55 ± 0.84 ^b^	3.56 ± 0.12 ^c^	11.98 ± 0.29 ^d^	24.46 ± 0.63 ^a^
1,2-Oxolinalool	12.983	0.92 ± 0.07	0.68 ± 0.06	0.4 ± 0.04		0.34 ± 0.07
n.i.	13.749		0.38 ± 0.07			0.24± 0.04
β—Linalool	14.122	1.51 ± 0.11 ^a^	0.73 ± 0.06 ^b^	0.26 ± 0.04 ^b^		
1-Octenyl acetate	14.413	3.32 ± 0.12 ^a^	0.37 ± 0.08 ^b^	0.28 ± 0.02 ^b^		0.79 ± 0.08 ^b^
n.i.	15.387			0.46 ± 0.03		
Camphor	15.957	3.17 ± 0.12 ^ab^	3.43 ± 0.10 ^a^	2.57 ± 0.09 ^b^	5.78 ± 0.14 ^c^	0.56 ± 0.07 ^d^
Borneol	16.907	9.6 ± 0.47 ^a^	9.36 ± 0.15 ^a^	6.25 ± 0.17 ^b^	13.9 ± 0.33 ^c^	1.4 ± 0.11 ^d^
n.i.	17.229			0.82 ± 0.07		
Cryptone	17.459			2.54 ± 0.12	1.79 ± 0.28	3.1 ± 0.11
Isobornyl formate *	19.047		0.97 ± 0.09	0.86 ± 0.07		
Linalyl acetate	19.794	2.47 ± 0.22 ^a^		0.28 ± 0.04 ^b^		0.13 ± 0.02 ^b^
Lavandulol	21.025	1.21 ± 0.07 ^a^		2.16 ± 0.17 ^b^	1.28 ± 0.11 ^a^	0.9 ± 0.07 ^a^
Isobornyl acetate	21.091					0.5 ± 0.06
n.i.	21.944			0.45 ± 0.04		0.2 ± 0.04
n.i.	23.706			0.19 ± 0.07		0.2 ± 0.05
cis-Geraniol *	24.393	2.79 ± 0.22 ^a^	0.4 ± 0.07 ^b^	1.44 ± 0.08 ^c^		1.07 ± 0.11 ^bc^
α- Santalene	25.833		1.97 ± 0.11 ^a^	2.77 ± 0.12 ^b^	1.61 ± 0.09 ^ac^	0.92 ± 0.07 ^c^
n.i.	26.301			0.24 ± 0.07		
γ-Cadinene	28.958			0.96 ± 0.08		0.15 ± 0.04
n.i.	28.968		0.22 ± 0.07			
n.i.	30.902		0.31 ± 0.08		0.18 ± 0.06	0.2 ± 0.05
n.i.	32.052			0.21 ± 0.04		
Total % of identified compounds		100	99.09	97.63	99.82	99.16

^1^ C—Control, SBE500—spruce bark extract at 500 mg dry bark/100 mL solvent, SBE1000—spruce bark extract at 1000 mg dry bark/100 mL solvent, BBE500—beech bark extract at 500 mg dry bark/100 mL solvent, BBE1000—beech bark extract at 1000 mg dry bark/100 mL solvent; n.i.—not identified, *—tentative identification. Different letters in the same row mean statistically significant differences at *p* < 0.05.
